# Risk of Adverse Clinical Outcomes in Hyponatremic Adult Patients Hospitalized for Acute Medical Conditions: A Population-Based Cohort Study

**DOI:** 10.1210/clinem/dgaa547

**Published:** 2020-08-20

**Authors:** Alexander Kutz, Fahim Ebrahimi, Soheila Aghlmandi, Ulrich Wagner, Miluska Bromley, Ben Illigens, Timo Siepmann, Philipp Schuetz, Beat Mueller, Mirjam Christ-Crain

**Affiliations:** 1 Endocrinology, Diabetes, and Metabolism, University Hospital Basel, Basel, Switzerland; 2 Endocrinology, Diabetes, and Metabolism, University Department of Medicine, Kantonsspital Aarau, Aarau, Switzerland; 3 General Internal and Emergency Medicine, University Department of Medicine, Kantonsspital Aarau, Aarau, Switzerland; 4 University Center for Gastrointestinal and Liver Diseases, Basel, Switzerland; 5 Basel Institute for Clinical Epidemiology and Biostatistics, Department of Clinical Research, University Hospital Basel, University of Basel, Basel, Switzerland; 6 Foundation National Institute for Cancer Epidemiology and Registration (NICER) University of Zurich, Zurich, Switzerland; 7 Center for Clinical Research and Management, Division of Health Care Sciences, Education Dresden, Dresden International University, Dresden, Germany; 8 Department of Neurology, Beth Israel Deaconess Medical Center, Harvard Medical School, Boston, MA, USA; 9 Department of Neurology, University Hospital Carl Gustav Carus, Technische Universität Dresden, Dresden, Germany; 10 Faculty of Medicine, University of Basel, Basel, Switzerland

**Keywords:** hyponatremia, SIAD, outcome, mortality, medical inpatients

## Abstract

**Context:**

Hyponatremia has been associated with excess long-term morbidity and mortality. However, effects during hospitalization are poorly studied.

**Objective:**

The objective of this work is to examine the association of hyponatremia with the risk of in-hospital mortality, 30-day readmission, and other short-term adverse events among medical inpatients.

**Design and Setting:**

A population-based cohort study was conducted using a Swiss claims database of medical inpatients from January 2012 to December 2017

**Patients:**

Hyponatremic patients were 1:1 propensity-score matched with normonatremic medical inpatients.

**Main Outcome Measure:**

The primary outcome was a composite of all-cause in-hospital mortality and 30-day hospital readmission. Secondary outcomes were intensive care unit (ICU) admission, intubation rate, length-of-hospital stay (LOS), and patient disposition after discharge.

**Results:**

After matching, 94 352 patients were included in the cohort. Among 47 176 patients with hyponatremia, 8383 (17.8%) reached the primary outcome compared with 7994 (17.0%) in the matched control group (odds ratio [OR] 1.06 [95% CI, 1.02-1.10], *P* = .001). Hyponatremic patients were more likely to be admitted to the ICU (OR 1.43 [95% CI, 1.37-1.50], *P* < .001), faced a 56% increase in prolonged LOS (95% CI, 1.52-1.60, *P* < .001), and were admitted more often to a postacute care facility (OR 1.38 [95% CI 1.34-1.42, *P* < .001). Of note, patients with the syndrome of inappropriate antidiuresis (SIAD) had lower in-hospital mortality (OR 0.67 [95% CI, 0.56-0.80], *P* < .001) as compared with matched normonatremic controls.

**Conclusion:**

In this study, hyponatremia was associated with increased risk of short-term adverse events, primarily driven by higher readmission rates, which was consistent among all outcomes except for decreased in-hospital mortality in SIAD patients.

A low plasma sodium level is the most prevalent electrolyte disorder encountered in hospitalized medical patients. Numerous studies have addressed the short- and long-term epidemiologic aspects of hospital associated hyponatremia, including its adverse effects on various patient-centered outcomes. While most of these studies have consistently reported a higher health care burden in patients with hyponatremia (1-3), some of them are limited by methodological drawbacks, such as insufficient adjustment for confounding by indication.

As randomized controlled trials are still lacking to assess the impact of hyponatremia and its treatment on clinical outcomes, a considerable number of retrospective studies have investigated its association (1, 4-8). Although most of these analyses were based on adjusted (including deidentified demographics, comorbidities, and severity indices) statistical models comparing patients with and without hyponatremia, baseline characteristics varied significantly. This unbalanced distribution of covariates led to a high risk of confounding by indication, since most hyponatremic patients were more severely ill than those without hyponatremia (9). Consequently, it is likely that adverse effects associated with hyponatremia per se may have suffered from considerable overestimation.

To overcome this important limitation, 2 previous studies used the method of propensity-score matching based on a variety of patient characteristics and thus providing better comparability between hyponatremic and nonhyponatremic patients (5, 10). While both studies found a higher health care burden in hospitalized hyponatremic patients including an increased length of hospital stay (LOS), a higher risk of intensive care unit (ICU) admission, a higher all-cause readmission rate, and higher overall costs, data on in-hospital mortality were not addressed.

Hence, the aim of this study was to assess the independent health-care burden of in-hospital hyponatremia among unselected propensity-score–matched adult medical inpatients.

## Methods

### Participants, data sources, and study variables

We conducted a population-based cohort study, using nationwide administrative claims data provided by the Swiss Federal Office for Statistics (Bundesamt für Statistik, Neuchâtel, Switzerland). This database provides deidentified individual-level data on patient demographics, health-care utilization, hospital typology, medical diagnoses, diagnostic tests, clinical procedures, and in-hospital patient outcomes for nearly all hospitalized patients in Switzerland. The database includes all Swiss inpatient discharge records from acute care-, general-, and specialty hospitals, excluding hospital units of postacute care institutions, regardless of payer, and thus, creates a near-complete sample of inpatient discharges in Switzerland between 2012 and 2017. No written informed consent was given by the patients, who were unidentifiable because of anonymization. Each hospitalization in this database was identified uniquely so that rehospitalizations could be tracked. A single patient may have more than one index admission in the study period. According to the Swiss “Diagnosis Related Groups” (SwissDRG) definition, all admissions after 18 days from discharge or admissions into another hospital were defined as a new case in the nationwide hospital claims data (11). For the end point of all-cause readmission rate, every rehospitalization within 30 days after discharge was counted as hospital readmission. Emergency and planned admissions to hospitals were both included in the analysis. Among patients who were transferred between acute care hospitals, the hospital stays were combined into a single episode of care and the patient outcome was attributed to the first hospitalization.

Medical diagnoses were coded using the International Classification of Diseases, version 10, German Modification (ICD-10 GM) codes (http://www.who.int/classifications/icd/en/). Cases with hyponatremia were identified by applying ICD-10-GM code E87.1 for hypoosmolar hyponatremia and E22.2 for syndrome of inappropriate antidiuresis (SIAD), respectively. Hyponatremia was defined based on sodium levels below 135 mmol/L or below 136 mmol/L, respectively, depending on the specific medical laboratory. The basis for coding hypoosmolar hyponatremia or SIAD was the discharge letter information.

Patients without a clear diagnosis of hypoosmolar hyponatremia were not included in the analysis to guarantee higher specificity. Nonmedical, psychiatric, and nonadult (age < 18 years) patients were excluded from the analysis. We also excluded geriatric patients from special geriatric units and ICU patients who were not treated on medical wards to achieve a higher specificity of the patient setting.

The institutional review board of Northwestern Switzerland approved this study and waived informed patient consent owing to the use of deidentified data. This study followed the STROBE (Strengthening The Reporting of Observational studies in Epidemiology) reporting guideline (12).

### Exposure

The exposure of interest was hypoosmolar hyponatremia in medical patients hospitalized for an emergency or planned clinical reason.

### Outcomes

The primary outcome was defined as a composite of all-cause in-hospital mortality and 30-day all-cause readmission. We have chosen this composite outcome because both end points are clinically relevant for patients and because it is in line with an ongoing randomized controlled trial investigating the impact of hyponatremia and its treatment on different patient outcomes (13). In addition, previous studies in the multimorbid and frail medical patient setting have also chosen such a composite outcome (14, 15), indicating the importance of measuring mortality and readmission rates in combination.

As secondary outcomes we assessed the individual components of the primary composite outcome, ICU admission rate, intubation rate, LOS (defined as days spent in the hospital during the hospitalization), and patient disposition after discharge. For disposition status, the event of interest was discharge to a short- or long-term postacute care facility or discharge back home. Based on previous findings with conflicting mortality rates among SIAD and non-SIAD hyponatremic patients in a smaller cohort (6), we explored the consistency of the findings in hyponatremia subgroups comparing patients with and without SIAD, respectively.

### Statistical analyses

Eligible hospitalizations according to inclusion and exclusion criteria were 1:1 propensity-score matched to a general medical inpatient control cohort (matched controls). The probability of being hyponatremic vs nonhyponatremic was calculated through a multivariable logistic regression model that contained all baseline covariates. The estimated propensity-score was used to match hyponatremic patients with nearest neighbor nonhyponatremic patients with a caliper size of 10^–9^ on the propensity scale. Covariate balance before and after propensity-score matching was assessed using standardized differences. A standardized difference of less than 10% was considered as an adequate balance between groups (16). For subgroup analyses, we performed separate propensity-score matchings within each study subcohort (SIAD and non-SIAD).

Estimates of the effect sizes and corresponding 95% CIs were determined using linear, logistic, or Cox proportional-hazards regressions as appropriate. Logistic regression analyses were used to estimate the overall association, Cox proportional-hazards regressions, however, assessed time-dependent 30-day estimates. All *P* values are 2-sided and have not been adjusted for multiple testing. Kaplan-Meier curves were used to illustrate differences in time to in-hospital mortality and readmission. All statistical analyses were performed using STATA, version 15.1 (StataCorp LLC).

## Results

### Patient characteristics

**Figure 1.**
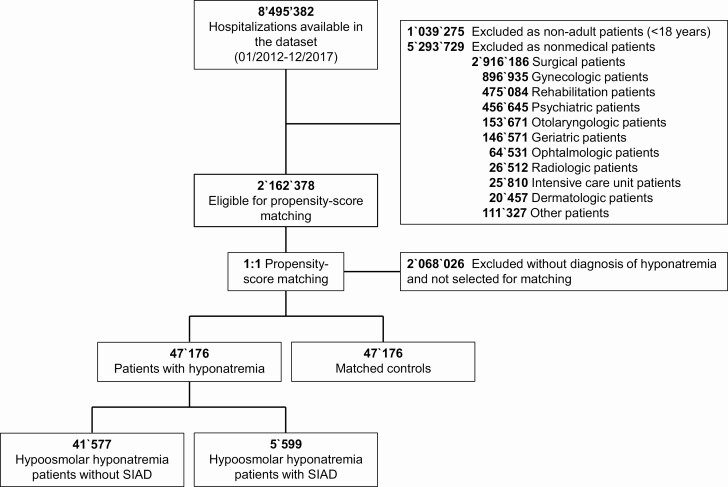
Flowchart of included study patients.

The database included 8 495 382 hospitalizations available between January 2012 and December 2017. After the exclusion of noneligible cases, 2 162 378 medical hospitalizations qualified for propensity-score matching. Of those, 47 176 hospitalized cases with hyponatremia were matched 1:1 with medical controls (Fig. 1). Baseline characteristics were comorbidities that were well balanced between both groups (Table 1) without any significant differences. Overall, mean age was 72.5 years, and 63.4% were female, the majority (78.8%) were treated in tertiary care hospitals, and almost all patients (97.4%) lived at home before hospital admission. Among hyponatremic patients, 11.9% had a coded diagnosis of SIAD. The main reasons for hospital admission were cardiovascular, oncologic, and pulmonary conditions.

**Table 1.** Baseline characteristics

	Hyponatremia vs matched controls		
	Patients with hyponatremia	Patients without hyponatremia	Standardized difference, %
Number of patients, n	47 176	47 176	
Sociodemographics			
Age, mean (SD)	72.5 (14.6)	72.5 (14.6)	0.02
Female sex, n (%)	29 904 (63.4)	29 913 (63.4)	0.04
Swiss residents, n (%)	43 626 (92.5)	43 625 (92.5)	0.01
Class of insurance, n (%)			
General	37 650 (79.8)	37 645 (79.8)	0.03
Semiprivate and private	9526 (20.2)	9531 (20.2)	0.03
Hospital teaching level, n (%)			
Tertiary care hospital	37 172 (78.8)	37 167 (78.8)	0.03
Living situation before admission, n (%)			
At home	45 961 (97.4)	45 948 (97.4)	0.30
Etiology of hyponatremia, n (%)			
Hyponatremia without SIAD	41 577 (88.1)	NA	NA
SIAD	5599 (11.9)	NA	NA
Main reasons for hospital admission, n (%)			
Endocrine	3206 (6.8)	3206 (6.8)	0
Infections	3319 (7.0)	3317 (7.0)	0.02
Cardiovascular	8436 (17.9)	8432 (17.9)	0.02
Cancer	5758 (12.2)	5768 (12.2)	0.06
Pulmonary	5905 (12.5)	5908 (12.5)	0.02
Comorbidities, n (%)			
Diabetes mellitus	5714 (12.1)	5700 (12.1)	0.09
Hypertension	25 863 (54.8)	25 856 (54.8)	0.03
Coronary artery disease	5089 (10.8)	5089 (10.8)	0
Heart failure	4688 (9.9)	4684 (9.9)	0.03
Cancer	7279 (15.4)	7289 (15.5)	0.06
Renal insufficiency	10 005 (21.2)	9990 (21.2)	0.08
Liver disease	1291 (2.7)	1291 (2.7)	0
Chronic obstructive pulmonary disease	3705 (7.9)	3703 (7.8)	0.02
Pneumonia	4356 (9.2)	4354 (9.2)	0.02
Charlson Comorbidity Index, mean (SD)	2.0 (2.7)	1.9 (2.5)	4.12

Abbreviations: NA, not applicable; SIAD, syndrome of inappropriate antidiuresis.

### Primary and secondary outcomes

The primary composite outcome occurred in 8383 (17.8%) hyponatremic patients and in 7994 (17.0%) medical controls, yielding an adjusted odds ratio (OR) of 1.06 (95% CI, 1.02-1.10, *P* = .001) (Table 2). Although there was no significant difference in all-cause in-hospital mortality between patients with and without hyponatremia, hyponatremic patients had a 7% increased risk for all-cause readmission within 30 days after hospital discharge (OR 1.07; 95% CI, 1.03-1.11, *P* = .001) (see Table 2).

**Table 2.** Primary and secondary outcomes

Patient outcomes	n (%)		OR (95% CI)	*P*
Primary outcome	Hyponatremic patients	Matched controls		
Composite outcome (all-cause in-hospital mortality and 30-d readmission)	8383 (17.8)	7994 (17.0)	1.06 (1.02-1.10)	.001
Secondary outcomes				
In-hospital mortality	2242 (4.8)	2200 (4.7)	1.02 (0.96-1.08)	.519
30-d readmission	6141 (13.0)	5794 (12.3)	1.07 (1.03-1.11)	.001
ICU admission	4506 (9.6)	3234 (6.9)	1.43 (1.37-1.50)	< .001
Intubation	1036 (2.2)	872 (1.9)	1.19 (1.09-1.31)	< .001
Length of hospital stay > 6 d	25 998 (55.1)	20 774 (44.0)	1.56 (1.52-1.60)	< .001
Discharge to postacute care facility	13 341 (29.7)	10 557 (23.5)	1.38 (1.34-1.42)	< .001

Abbreviations: OR, odds ratio; ICU, intensive care unit.

Compared with matched controls, patients with prevalent hyponatremia had a higher risk of ICU admission (OR 1.43; 95% CI, 1.37-1.50, *P* < .001) as well as for intubation OR 1.19; 95% CI, 1.09-1.31, *P* < .001). Furthermore, compared to propensity-score matched controls, patients with hyponatremia were hospitalized longer (OR 1.56; 95% CI, 1.52-1.60, *P* < .001) and had an increased risk of discharge to a postacute care facility (OR 1.38; 95% CI, 1.34-1.42, *P* < .001) (see Table 2).

### Comparison of syndrome of inappropriate antidiuresis vs non–syndrome of inappropriate antidiuresis causes of hyponatremia

**Figure 2.**
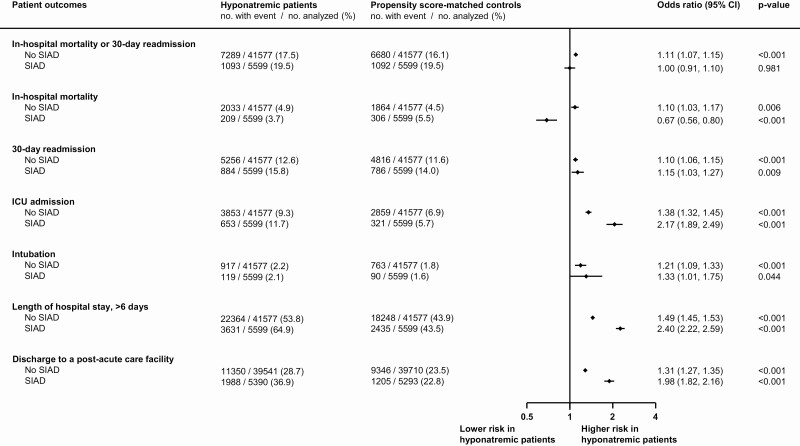
Risk comparison for primary and secondary outcomes stratified by non-SIAD and SIAD patients. ICU, intensive care unit; SIAD, syndrome of inappropriate antidiuresis.

**Figure 3.**
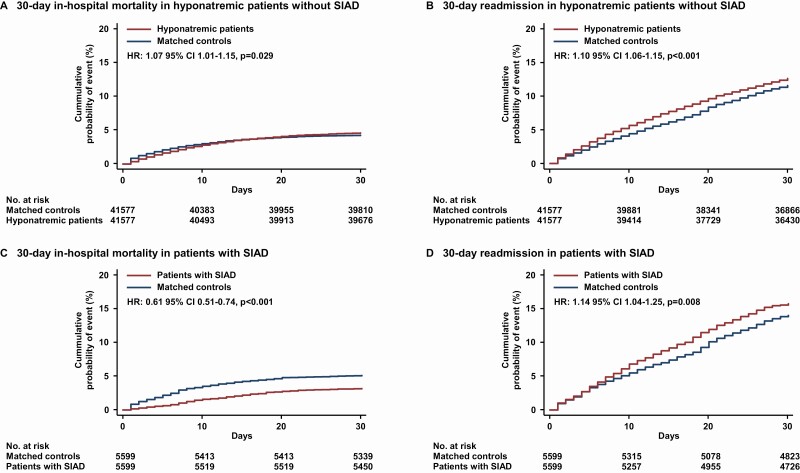
Kaplan-Meier analyses of all-cause in-hospital 30-day mortality and 30-day readmission stratified by non-SIAD and SIAD patients. Shown are estimates of the probability of a first occurrence of all-cause in-hospital mortality in A, non-SIAD and C, SIAD patients and 30-day readmission in B, non-SIAD and D, SIAD patients, respectively. Hazard ratios are based on Cox regression analyses. SIAD, syndrome of inappropriate antidiuresis.

To assess for consistency among SIAD and non-SIAD patients, we performed subgroup sensitivity analyses stratified by etiology of hyponatremia. In general, patients with SIAD were more severely ill as compared with non-SIAD patients, shown by a higher mean Charlson Comorbidity Index (2.4 vs 2.0 points, *P* < .001). While hyponatremic patients with hyponatremia caused by conditions other than SIAD were at increased risk for primary and secondary outcomes, patients with SIAD were at decreased risk of in-hospital mortality (OR 0.67; 95% CI, 0.56-0.80, *P* < .001) when compared to their matched medical controls (Figs. 2C and 3C).

Figure 3 shows the Kaplan-Meier cumulative incidences of all-cause in-hospital mortality and all-cause readmission within 30 days stratified by non-SIAD and SIAD patients. During 30-day follow-up, the hazard ratios, comparing non-SIAD with their respective matched controls, were 1.07 (95% CI, 1.01-1.15, *P* = .029) for in-hospital mortality and 1.10 (95% CI, 1.06-1.15, *P* < .001) for 30-day readmission. Comparing SIAD patients with their corresponding matched control population, the hazard ratio for in-hospital mortality was 0.61 (95% CI, 0.51-0.74, *P* < .001) and for 30-day readmission 1.14 (95% CI, 1.04-1.25, *P* = .008). However, patients with SIAD likewise were at significantly higher excess risks for ICU admission, prolonged LOS, and discharge to postacute care facilities (see Fig. 2).

## Discussion

This large population-based cohort study investigating the association of hyponatremia and adverse outcomes among hospitalized medical in-patients revealed two key findings: Hyponatremia—in general—was independently associated with more events of in-hospital mortality or 30-day readmission, ICU admission, intubation, as well as prolongation of LOS, and higher risk of discharge to a postacute care facility. Interestingly, the etiology of hyponatremia had fundamental implications for its association with in-hospital mortality, with significantly lower mortality among patients with alleged SIAD.

In this unselected medical population of hospitalized patients using real-world data, hyponatremia was associated with increased in-hospital health-care utilization. The conclusion of this study is consistent with previous findings showing that in-hospital mortality and readmission rates are higher in hyponatremic patients (5, 7) and that this vulnerable patient population is more likely to be admitted to the ICU (17), has an increased LOS, and a higher probability of being discharged to a postacute care facility (8), or being readmitted to the hospital (10), respectively.

The presence of hyponatremia has consistently been associated with higher mortality compared with patients without hyponatremia (18). However, published mortality rates vary significantly, spanning from 1.5- to 60-fold increases compared to nonhyponatremic patients (18-20). Given this broad spectrum of observed excess risk, confounding factors beyond disturbances of sodium levels are likely to influence this association. Besides diverging clinical settings and varying definitions of hyponatremia, most studies lack methodologic robustness, leading to strong deviations between univariable and multivariable analyses (6, 21).

As a consequence, it is still a matter of debate whether hyponatremia per se promotes increased mortality (8, 22, 23), or whether it is rather a surrogate of the severity of the underlying disease (9, 24-26). Thus, the increased risk of mortality found in several observational studies might result from a complex interaction of various patient- and setting-related factors (27-29). Therefore, randomized controlled trials are urgently needed to provide the missing evidence and to demonstrate whether an active sodium correction will result in a favorable patient outcome (13).

Apart from mortality, several studies have explored the incremental risk of several other clinical complications associated with hyponatremia. In patients with heart failure, kidney disease, liver cirrhosis, as well as in general medical inpatients, hyponatremia was found to be associated with increased LOS (8, 30) and risk for rehospitalization (10). Wald et al. observed higher rates of discharge to a short- or long-term care facility (8), similar to our findings.

Two previous studies have also performed propensity-score matched analyses to better address methodologic limitations (5, 10). They found hyponatremia to be consistently associated with increased LOS, 30-day readmission rates, and hospital costs. In contrast to analyses, findings from these propensity-score matched studies consistently reported less impressive associations of hyponatremia with clinical outcomes. In line, we found a moderately increased risk for 30-day readmission (+7%), and a more excessive risk for ICU admission (+43%), intubation (+19%), prolonged LOS (+56%), and discharge to a postacute care facility (+38%) compared with matched medical controls.

Interestingly, we did not observe an increase in in-hospital mortality in general, but sensitivity stratification by non-SIAD and SIAD hyponatremic patients showed opposing results. While hyponatremic patients without underlying SIAD had a 10% higher risk for in-hospital mortality, the development of SIAD seemed indeed to be associated with reduced mortality. There are only a few data on mortality in patients with SIAD-induced hyponatremia. One recent prospective observational study showed, in contrast to our data, higher mortality rates in patients with SIAD as compared with normonatremic controls (4), even though event rates were lower as compared with non-SIAD hyponatremic patients. However, these Irish data were not adjusted for important baseline characteristics and SIAD patients had significantly more malignant (28 vs 19%) and metastatic (21 vs 13%) comorbidities as compared with normonatremic ones.

Potential reasons for a possible “protective” role of SIAD are unclear and speculative. It has been shown that paradoxically, mortality rate seems to be lower in patients with pronounced hyponatremia (below 120 mmol/L) as compared to patients with a milder hyponatremia (4, 6). Interestingly, the degree of hyponatremia varies according to hyponatremia etiology and notably, SIAD seems to predispose to very low sodium levels (6). In patients with sodium levels below 120 mmol/L, the therapeutic approach has been shown to be different, with a higher percentage of ICU admission and of hypertonic saline administration as well as a more rapid initial sodium correction rate (6, 28). This could possibly explain the lower mortality rates but higher ICU admission rates in these patients. However, first, we do not have sufficient evidence that severe hyponatremia is more prevalent among SIAD patients and, second, we do not have exact sodium levels in our database to further underline this hypothesis. An alternative explanation for the “protective” role of SIAD might be the exact opposite, that is, that patients with SIAD had more mild hyponatremia. However, taking into account that hyponatremia awareness among physicians is low (31-33) and that hyponatremia is therefore underreported in ICD-10 codes, we assume that mostly the severe cases, rather than the mild ones, were reported. Finally, we cannot definitively exclude that other essential factors that are more difficult to perceive and measure (such as the severity of the intercurrent diseases that led patients to hospitalization, the large spectrum of medical complications encountered by patients across hospitalization, or the proportion of patients receiving palliative care) might not have been distributed in a comparable way in both groups. Therefore, prospective interventional randomized trials are needed to prove a causal role of hyponatremia due to SIAD in patient prognosis (3).

We found robust results in almost 5600 SIAD patients, leading to an alternative, more hypothetical explanation that (over-)production of the antidiuretic hormone (vasopressin) during acute medical conditions might be an *appropriate* sign of immediate hormonal response. It is well established that various stressors activate the hypothalamic-pituitary-adrenal axis, increasing vasopressin secretion. Thus, hyponatremia may simply be a marker for high levels of a stress hormone to maintain blood pressure and preserve fluid volume, an evolutionary advantage in “flight-or-fight” situations. Therefore, we may postulate that patients showing an adequate high vasopressin response in a “stress” situation have a survival advantage compared to comparably ill patients without such a capability. However, whether the increase in antidiuretic hormones is *appropriate* or *inappropriate* warrants further elucidation.

The major strength of this study is its high external validity, which is based on the large nationwide sample comprising nearly 100 000 patients. Within this cohort, weighted analysis allowed for highly accurate estimates at the national level.

Our data must be interpreted in the context of the study design. First, a certain risk of misclassification and underreporting needs to be acknowledged because administrative data were used in our analyses and we were not able to ascertain the diagnoses. Second, in our study population, merely 2.2% of all included hospitalized medical patients were diagnosed with hyponatremia and qualified for propensity-score matching, although current evidence suggests a much higher prevalence among hospitalized patients (7, 8, 34, 35). Nevertheless, we were able to include more than 47 000 patients into our propensity-score matched analysis of real-world data. This rather strong data basis, in conjunction with the consistency of the observed effects of hyponatremia on clinical outcome measures in this study, indicates a high degree of internal validity and generalizability. Third, the diagnosis of hyponatremia was based on ICD-10 classification in discharge reports and in some of these reports non-SIAD and SIAD causes of hyponatremia were not distinguished. Because we do not have information on clinical symptoms and severity of hyponatremia, we are unable to account for unmeasured and unmeasurable residual confounding (eg, etiology of hyponatremia, thirst perception, volume status, body mass index, smoking status).

In conclusion, there is a relevant health-care burden among hyponatremic adult patients hospitalized for acute medical conditions. Further studies are needed to investigate underlying mechanisms on how hyponatremia may differentially modulate clinical outcomes.

## Data Availability

Restrictions apply to the availability of data analyzed during this study because they were used under license. The corresponding author will on request detail the restrictions and any conditions under which access to some data may be provided.

## Abbreviations

ICU: intensive care unit
LOS: length of hospital stay
SIAD: syndrome of inappropriate antidiuresis

## Additional Information

***Disclosure Summary:*** The authors have nothing to disclose.
